# Reduced glymphatic function in autism revealed by the diffusion tensor analysis along the perivascular space index

**DOI:** 10.1007/s00702-026-03249-2

**Published:** 2026-08-07

**Authors:** Laust Vind Knudsen, Manouchehr Seyedi Vafaee, Abigail Jane Sheldrick-Michel, Tanja Maria Michel

**Affiliations:** Psychiatric Research unit, Odense University Hospital, University of Southern Denmark, Odense, Denmark

**Keywords:** Autism, ASD, Glymphatic system, ALPS-index, Diffusion tensor imaging, MRI

## Abstract

The glymphatic system supports brain homeostasis by clearing interstitial solutes via perivascular pathways. Alterations in glymphatic function have been linked to neurodevelopmental and neurodegenerative disorders, but its role in autism spectrum disorder (ASD) remains unclear. We analyzed diffusion tensor imaging (DTI) magnetic resonance imaging data from five cohorts in the Autism Brain Imaging Data Exchange (ABIDE) project. Glymphatic function was estimated using the DTI analysis along the perivascular space (ALPS) index. ASD diagnoses were confirmed according to DSM-IV-TR or DSM-5 criteria, and neurotypical (NT) participants had no history of neurological, psychiatric, or developmental disorders. The final sample comprised 250 participants (128 ASD, mean age = 18.57 ± 13.81; 122 NT, mean age = 21.32 ± 14.37). Two-way analysis of variance (ANOVA) revealed a significant main effect of diagnosis, F(1, 246) = 10.44, *p* = 0.0014, with lower ALPS-indices in ASD. A diagnosis-by-age interaction was also observed, F(1, 246) = 4.71, *p* = 0.031. Post hoc tests demonstrated that autistic adults (≥ 18 years) had significantly lower ALPS-indices than NT adults (*p* = 0.0004), whereas no group differences were demonstrated between younger ASD and NT (< 18 years). In ASD adults, the ALPS-index correlated negatively with depressive symptoms (*r* = − 0.489, *p* = 0.013), but not with IQ or autistic traits. These findings suggest that glymphatic dysfunction in ASD may follow a developmental trajectory, with alterations becoming most evident in adulthood, potentially contributing to an increased risk of developing neurodegenerative disorder in autistic individuals.

**Supplementary Information:**

The online version contains supplementary material available at https://doi.org/10.1007/s00702-026-03249-2.

## Introduction

Autism spectrum disorder (ASD) is a neurodevelopmental condition affecting more than 2.3% of 8-year-old children (Maenner et al. 2021). ASD is characterized by difficulties in social interaction and communication, along with restricted, repetitive behaviors and atypical sensory processing (American Psychiatric Association 2013). In addition to these core features, sleep disturbances are highly prevalent in children with ASD, with reported rates ranging from 50% to 80%, compared to 9% to 50% in neurotypical (NT) children (Richdale and Schreck 2009; Burman et al. 2023). Furthermore, sleep-related problems often persist into adulthood, with some studies reporting that up to 86% of autistic adults experience at least one form of sleep disturbance (Lampinen et al. 2022). The most frequently reported sleep disturbances include difficulties initiating and maintaining sleep, as reported subjectively, and objectively measured patterns such as prolonged sleep onset latency, reduced total sleep duration, and decreased sleep efficiency (Lampinen et al. 2022; Burman et al. 2023). These sleep disturbances are associated with impairments in daytime functioning, increased behavioral challenges, and cognitive deficits, particularly in children with ASD (Carnett et al. 2021). Moreover, ASD is coupled with higher rates of depression and anxiety (Hudson et al. 2019), both of which have been linked to sleep disturbances in autistic individuals (Stewart et al. 2020; Puzino et al. 2021).

**Fig. 1:**
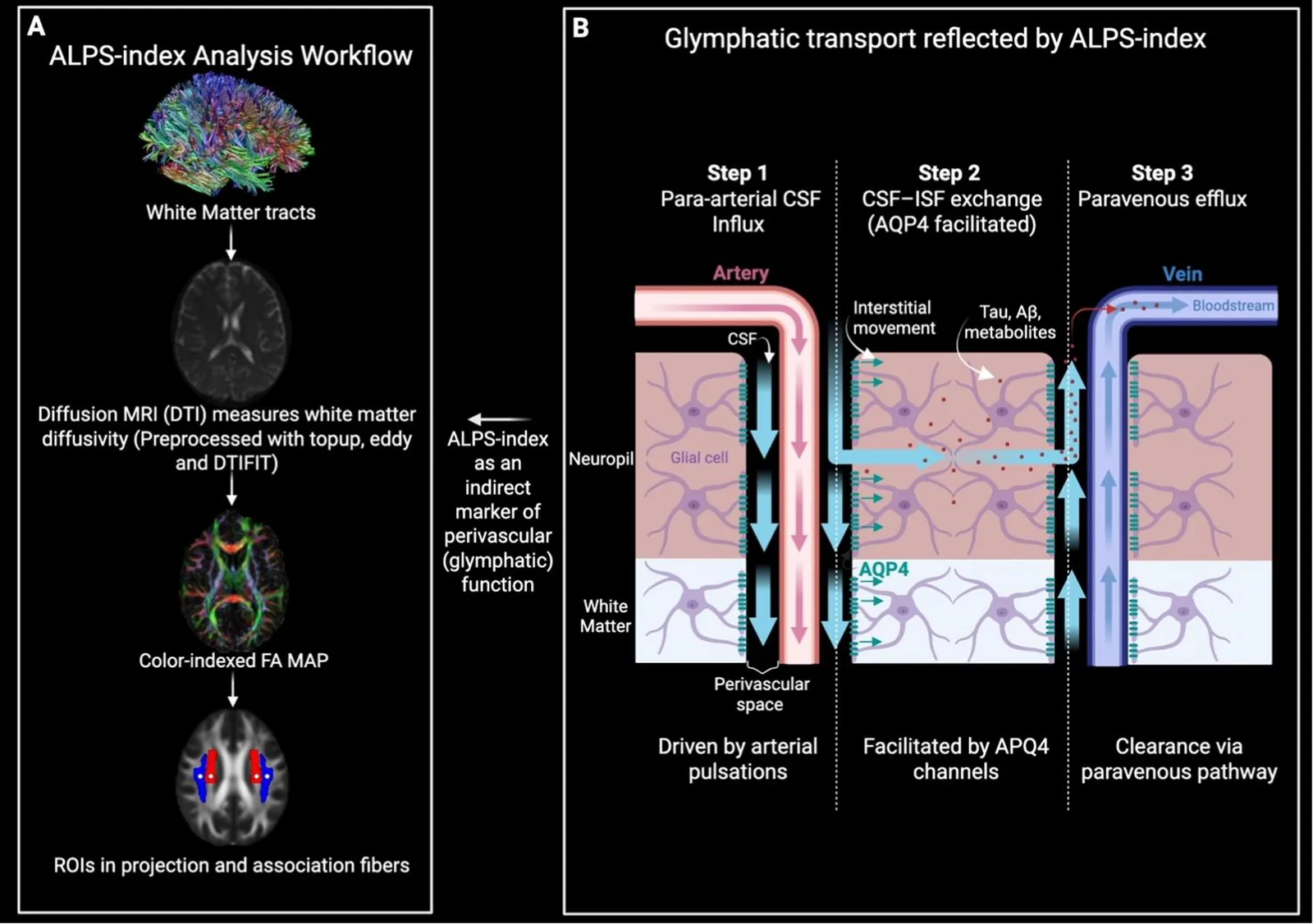
Workflow of ALPS-index Calculation and its Relation to Glymphatic Transport. **A** Diffusion MRI data were preprocessed (topup, eddy, and DTIFIT) to generate fractional anisotropy (FA) maps. At the level of the body of the lateral ventricle, regions of interest (ROIs) were placed in projection and association fibers to derive diffusivity measures along the x-, y-, and z-axes. These measures were used to calculate the ALPS-index, providing an indirect marker of perivascular diffusivity. **B** Schematic representation of glymphatic transport, which the ALPS-index is thought to reflect. CSF enters the brain parenchyma along para-arterial perivascular spaces (Step 1), mixes with interstitial fluid to facilitate solute clearance (Step 2, mediated by AQP4 water channels on astrocytic endfeet), and drains along paravenous pathways into the bloodstream (Step 3). Aβ = amyloid-β. AQP4 = aquaporin-4. CSF = cerebrospinal fluid. ISF = interstitial fluid

Beyond its effects on cognitive abilities and mental health, sleep plays a critical role in clearing metabolic waste from the brain through the glymphatic system (Iliff et al. 2012, 2013). The glymphatic system facilitates clearance of metabolic waste from the brain by directing cerebrospinal fluid (CSF) into the parenchyma along specialized perivascular spaces (PVSs) that surround cerebral blood vessels (Fig. 1). These spaces are bordered by astrocytic endfeet, which are densely enriched with aquaporin-4 (AQP4) water channels and form the structural barrier known as the glia limitans. Through this arrangement, CSF entering along arterial PVSs mixes with interstitial fluid (ISF), promoting solute exchange and supporting the removal of neurotoxic proteins and other metabolites. The mixture is then directed toward venous PVSs for clearance from the brain, making AQP4-mediated water transport at astrocytic endfeet a key driver of glymphatic function. Impaired glymphatic function has been associated with reduced cognitive performance (Kamagata et al. 2022), whereas an efficient glymphatic system may protect against age-related cognitive decline (Wang et al. 2023). This link between glymphatic activity and cognition is thought to be partially mediated by the accumulation of neurotoxic proteins, such as amyloid-β and tau neurofibrillary tangles, which are key neuropathological hallmarks of Alzheimer’s disease (AD) (Binette et al. 2022). Indeed, amyloid-β accumulation has been linked to impaired glymphatic function in both rodents (Iliff et al. 2013; Harrison et al. 2020) and humans (Park et al. 2023; Okazawa et al. 2024). Moreover, the role of the glymphatic system in clearance of amyloid-β and tau has been well established in rodents (Iliff et al. 2012).

Consequently, reduced glymphatic function may play a role in age-related cognitive decline and neurodegeneration, potentially facilitating brain atrophy and the development of AD. Notably, although ASD is a neurodevelopmental disorder, it persists throughout life and has recently been linked with a 2.6-fold increased risk of developing AD (Vivanti et al. 2021). Supporting this, postmortem studies have reported significantly greater amyloid-β accumulation in autistic individuals compared to NT individuals (Wegiel et al. 2012). These findings suggest that impaired glymphatic function may contribute to the elevated risk of AD observed in ASD (Vivanti et al. 2021). Evidence suggests that glymphatic dysfunction in ASD may begin early in life. Studies report excessive CSF in the subarachnoid space surrounding the cortical surface, thought to reflect impaired CSF circulation, in autistic children as early as 6 months and up to 3 years of age (Shen et al. 2018; Garic et al. 2023). Enlarged PVS at 24 months have been linked to a 2.2-fold higher likelihood of ASD diagnosis and an increased risk of sleep problems by the age of 8 years (Garic et al. 2023). Such early alterations support the view that impaired glymphatic function may represent a core component of ASD neuropathology.

Initially, human glymphatic function was assessed using invasive intrathecal injections of gadolinium, a technique referred to as glymphatic imaging. More recently, non-invasive methods such as diffusion tensor image (DTI) analysis along the perivascular space (ALPS-index) have been developed (Taoka et al. 2017). The ALPS-index utilizes diffusion imaging to quantify water diffusivity along perivascular spaces, thereby serving as an indirect proxy of glymphatic function. Glymphatic activity is believed to depend strongly on water movement within these spaces, particularly in regions adjacent to the lateral ventricles. Accordingly, the ALPS-index is derived from diffusivity in the radial direction (Fig. 1), which represents the most direct route for solute clearance from the ventricular bodies toward the brain surface or ventricular walls (Taoka et al. 2017, 2024). Histological changes within the glymphatic pathway are expected to alter water diffusivity, especially along the right-left axis. Thus, the ALPS-index offers a highly reproducible (Liu et al. 2024) non-invasive alternative to gadolinium-based glymphatic imaging (Taoka et al. 2024), with the two methodologies demonstrating strong correlation (Zhang et al. 2021).

Despite the well-established association between ASD and sleep disturbances (Lampinen et al. 2022; Burman et al. 2023), the increased risk of AD (Vivanti et al. 2021), and evidence of glymphatic abnormalities in autistic infants and young children (Shen et al. 2018; Garic et al. 2023), only a few studies to date have examined the ALPS-index in autistic individuals. All reported significantly lower ALPS-index values in autistic children aged 2–7 years compared to NT children (Li et al. 2022; Wang et al. 2025; Tian et al. 2025), suggesting reduced glymphatic function in this population.

Because previous investigations were limited to young children and modest sample sizes, the present study aimed to: (1) test whether ALPS-index is reduced in autistic compared to NT individuals, (2) examine whether this reduction is present in autistic adults, and (3) evaluate whether prior reports of reduced ALPS-index in autistic children could be replicated in a larger sample.

## Methods

Given the limited number of studies applying the ALPS-index in autistic individuals, and that the existing studies focused exclusively on young children (< 7 years), we did not restrict participant age. Instead, participants were stratified into two groups: youth (< 18 years) and adults (≥ 18 years), enabling evaluation of ALPS-index across age ranges not previously examined.

### Participants

Data for the present study were obtained from the publicly available Autism Brain Imaging Data Exchange (ABIDE) repository (Di Martino et al. 2014). Sites were included if their datasets contained diffusion MRI scans for both autistic and neurotypical participants. This resulted in the inclusion of five cohorts: Barrow Neurological Institute (*n* = 58), Institute Pasteur and Robert Debré Hospital (*n* = 56), NYU Langone Medical Center – Sample 1 (*n* = 78), Trinity Centre for Health Sciences (*n* = 42), and San Diego State University (*n* = 58).

**Fig. 2:**
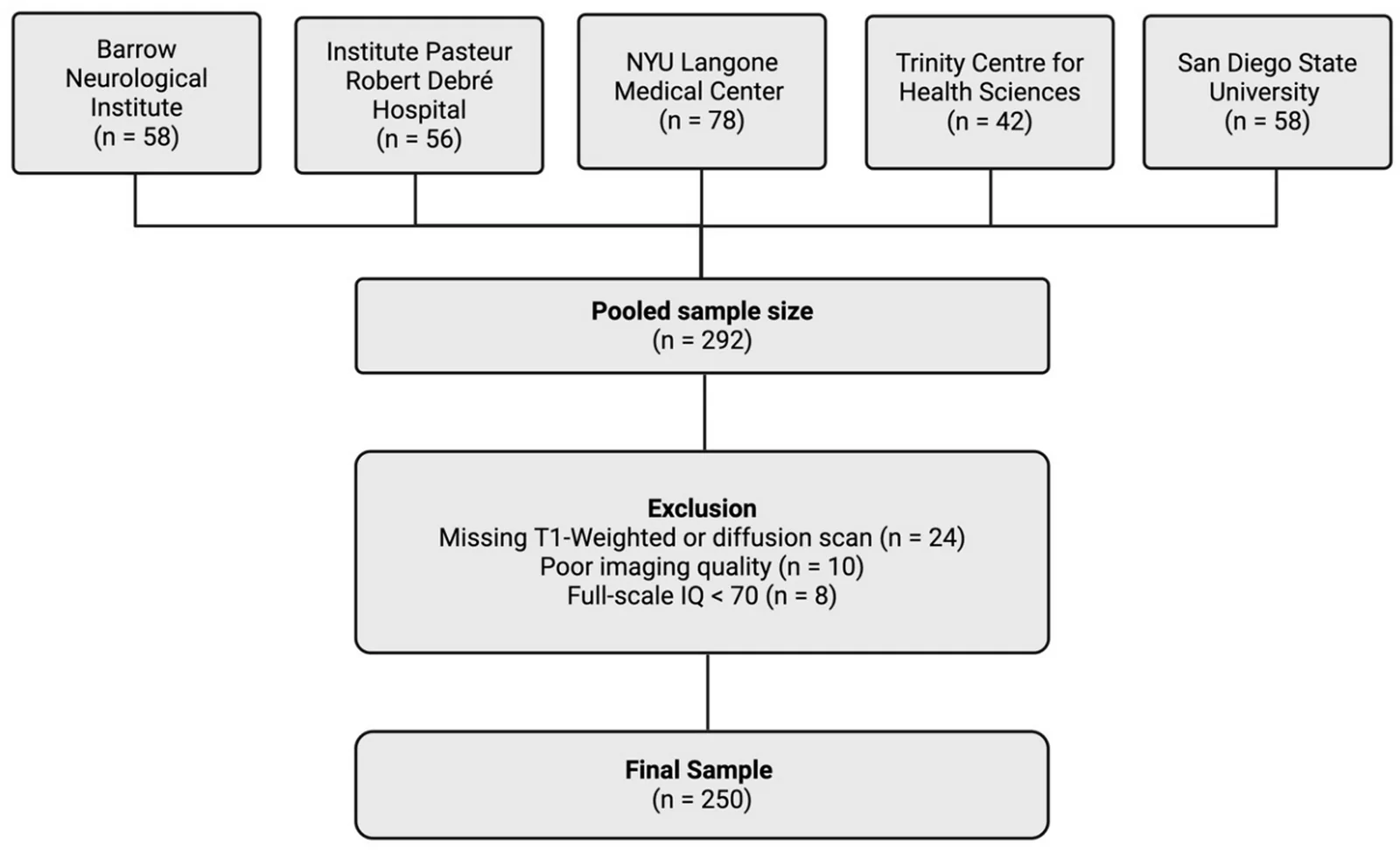
Participant Inclusion and Exclusion Flowchart. The flowchart illustrates the inclusion and exclusion process of participants across cohorts, leading to the final study sample

Participants in the ASD group had a prior clinical diagnosis of ASD, which was confirmed at each site using gold-standard diagnostic instruments, including the Autism Diagnostic Observation Schedule (ADOS) and/or the Autism Diagnostic Interview–Revised (ADI-R), in accordance with DSM-IV-TR or DSM-5 criteria (American Psychiatric Association 2013). NT participants had no history of neurological, psychiatric, or developmental disorders. Exclusion criteria included: (1) missing T1-weighted or diffusion-weighted images, (2) full-scale IQ < 70 when available, and (3) poor imaging quality, determined through visual inspection and site-specific quality control procedures. After applying these criteria, the final sample comprised 250 participants: 128 with ASD and 122 NT (Fig. 2). All MRI data were acquired on 3 Tesla scanners. Detailed information on scanner models, sequence parameters, and site-specific acquisition protocols is available on the ABIDE project website (https://fcon_1000.projects.nitrc.org/indi/abide/).

### Diffusion tensor imaging preprocessing

The 4D DTI datasets were converted from DICOM to NIfTI format using *dcm2niix* (Li et al. 2010). Image preprocessing included artifact correction via the Marchenko-Pastur principal component analysis (MP-PCA) denoising algorithm and Gibbs ringing removal, implemented with the Mrtrix3 (Tournier et al. 2019) commands dwidenoise and mrdegibbs, respectively. Susceptibility-induced distortion, eddy current, and motion corrections were performed using the FMRIB Software Library (FSL) version 6.0.1 commands *topup* and *eddy* (Andersson et al. 2003). Fractional anisotropy (FA) maps and diffusivity maps along the x-, y-, and z-axes were generated with the FSL command *dtifit* (Jenkinson et al. 2012). Each subject’s FA map was co-registered to the JHU-ICBM-FA template in FSL, and the resulting transformation matrix was applied to all diffusivity maps using the FSL command flirt.

### ALPS-index

The ALPS-index was used to get an indirect measure of glymphatic function following the methods described by Taoka et al. (2017). This approach quantifies water diffusivity along the PVS at the level of the lateral ventricle bodies, where perivascular flow is oriented predominantly along the right-left axis, corresponding to the x-axis in the diffusion image (Fig. 1). Diffusivity coefficients were extracted from two major fiber systems traversing this region: projection fibers adjacent to the ventricular wall (craniocaudal, z-axis) and association fibers located more laterally (anteroposterior, y-axis). The x-axis diffusivity in projection (Dx_proj) and association (Dx_assoc) fiber ROIs was normalized by the y-axis diffusivity in projection fibers (Dy_proj) and z-axis diffusivity in association fibers (Dz_assoc). We utilized an automatic pipeline to reduce subjectivity in placement of the regions of interest (ROIs) as described by Zhang et al. (2021). ROIs (5 mm diameter) were defined using an atlas-based approach (ICBM-DTI-81 template) (Hua et al. 2008) and restricted to regions perpendicular to the lateral ventricles. Voxels with FA ≤ 0.2 were excluded to avoid cerebrospinal fluid contamination.

The ALPS-index was determined according to previously described methods (Taoka et al. 2017). Specifically, it was calculated as:

$${\mathbf{ALPS}} - {\mathbf{index}}=\frac{{{\mathrm{mean}}\left( {{\mathrm{Dx}}.{\mathrm{proj}}+{\mathrm{~Dx}}.{\mathrm{assoc}}} \right)}}{{{\mathrm{mean}}\left( {{\mathrm{Dy}}.{\mathrm{proj}}+{\mathrm{~Dz}}.{\mathrm{assoc}}} \right)}}$$

The computation was performed separately for the left and right hemispheres. The resulting values were then averaged to obtain a single bilateral ALPS-index, which was subsequently used for statistical analyses.

### Statistical analysis

All analyses were performed in R (version 2025.05.1 + 513). Continuous variables were summarized as means and standard deviations, and group differences in demographic and clinical characteristics between ASD and NT participants were assessed using independent-samples *t*-tests. Categorical variables were compared using χ² tests. The primary analysis examined whether the ALPS-index differed by diagnosis (ASD vs. NT), by age group (younger participants < 18 years vs. adults ≥ 18 years), and by their interaction, using a two-way analysis of variance (ANOVA). Partial eta squared (ηp²) with 95% confidence intervals (CI) was calculated as an effect size measure for the ANOVA. When significant main effects or interactions were detected, we performed post hoc pairwise comparisons using estimated marginal means (*emmeans*), applying Bonferroni adjustments to control for multiple comparisons within each family of tests. Cohen’s *d* was reported for effect size of post hoc tests. Due to the high reproducibility of the ALPS-index, even between scanner vendors (Liu et al. 2024) we did not correct for scanner albeit the use of data acquired from different scanners. However, a cohort-wise comparison is available in Supporting Information Table S1 and Fig. S1. Associations between ALPS-index and continuous clinical or cognitive measures were examined using Pearson correlation coefficients (*r*), computed in the whole sample, and stratified by diagnosis and age group. Correlations were performed for Full-scale Intelligence Quotient (FIQ), ADOS total scores, and Beck Depression Inventory (BDI) scores (BDI was assessed only in the Barrow Neurological Institute cohort). Two-tailed p*-*values < 0.05 were considered statistically significant. Effect sizes for correlations were interpreted according to conventional benchmarks (small: |*r*| ≥ 0.10, medium: |*r*| ≥ 0.30, large: |*r*| ≥ 0.50).

## Results

**Fig. 3:**
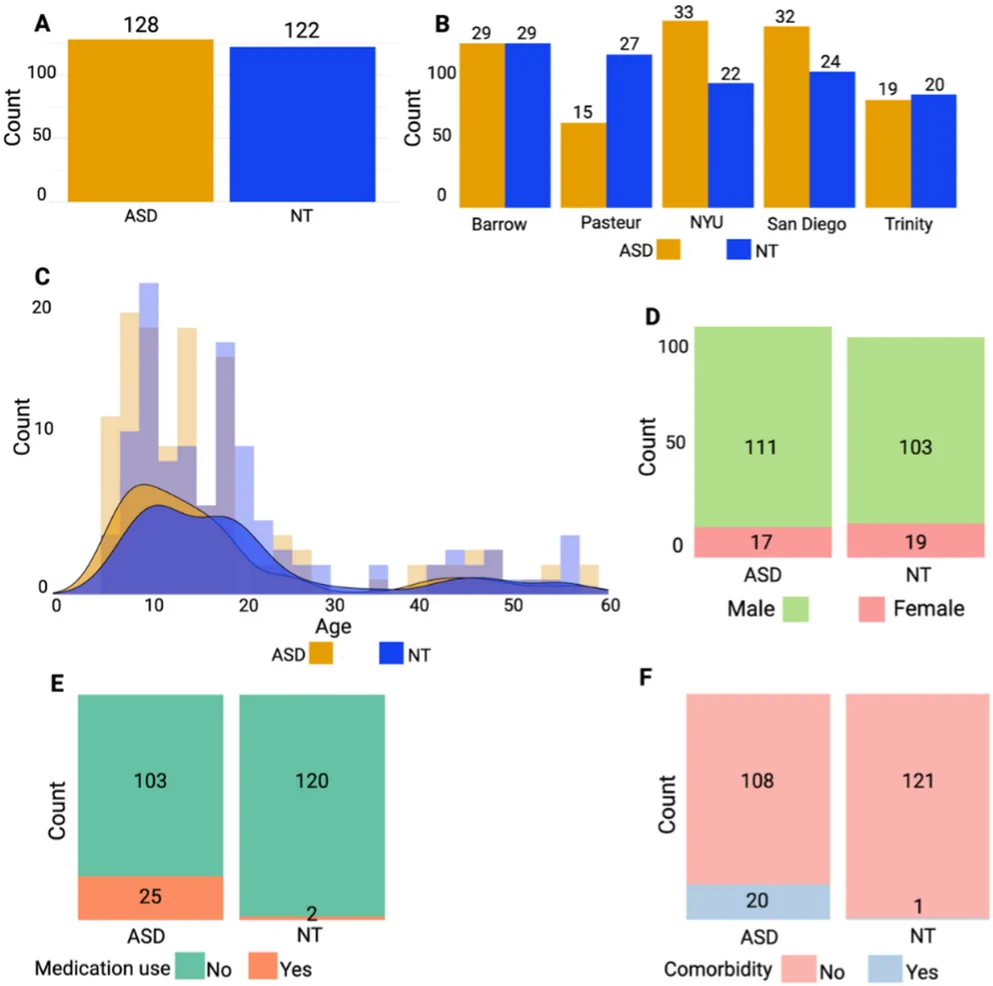
Overview of The Study Participant and Cohort Characteristics. **A** Number of participants in each diagnostic group (ASD and NT). **B** Distribution of participants across the five contributing cohorts. **C** Age distribution of all included participants. **D** Sex distribution across diagnostic groups. **E** Proportion of participants using medication. **F** Proportion of participants with comorbid conditions

The final sample comprised 250 participants, including 128 autistic and 122 NT individuals (Fig. 3). No significant differences were observed between the ASD and NT groups in terms of age or gender distribution, either in the overall sample or when stratified by age (< 18 vs. ≥18 years). However, the ASD group exhibited significantly higher proportions of comorbidity and medicine use, as well as lower FIQ scores and higher BDI scores compared to the NT group (Table 1).

**Table 1:** Demographic and Clinical Characteristics of The ASD and NT Group

Variable	Group	ASD			NT			Analysis		
		*N*	Mean	SD	*N*	Mean	SD	t	df	*p*
Age (years)	Whole sample	128	18.57	13.81	122	21.32	14.37	1.538	246	0.125
	<18 years	86	11.25	3.49	65	11.88	3.19	1.394	143.5	0.166
	≥18 years	42	33.83	14.51	57	32.08	14.61	0.594	88.8	0.554
		*N*	*N*	*%*	*N*	*N*	*%*	*X* ^**2**^	*df*	*p*
Female	Whole sample	128	16	12.50	122	19	15.57	0.489	1	0.484
	<18 years	86	11	12.79	65	7	10.77	0.149	1	0.699
	≥18 years	42	5	11.90	57	12	21.05	1.421	1	0.233
		*N*	*N*	*%*	*N*	*N*	*%*	*X* ^**2**^	*df*	*p*
Comorbidity	Whole sample	128	19	14.84	122	1	0.82	14.841	1	>0.001
	<18 years	86	16	12.79	65	1	1.54	7.358	1	0.007
	≥18 years	42	3	7.14	57	0	0	2.112	1	0.145
		*N*	*N*	*%*	*N*	*N*	*%*	*X* ^**2**^	*df*	*p*
Medicine use	Whole sample	128	25	19.53	122	2	1.64	19.307	1	>0.001
	<18 years	86	17	19.76	65	1	1.54	10.232	1	0.001
	≥18 years	42	8	19.05	57	1	1.75	6.936	1	0.008

FIQ		*N*	Mean	SD	*N*	Mean	SD	t	df	*p*
	Whole sample	123	104.8	15.70	99	111.6	14.45	3.353	216.1	0.001
	<18 years	82	103.7	16.55	62	110.6	16.07	2.526	133	0.013
	≥18 years	41	107.1	13.73	37	113.32	11.21	2.202	75.2	0.031
ADOS	Whole sample		12.17	4.53						
	<18 years		12.38	4.79						
	≥18 years		11.32	3.73						
BDI	Whole sample	25	11.22	10.09	29	5.12	6.50	2.595	39.8	0.013
	<18 years									
	≥18 years	25	11.22	10.09	29	5.12	6.50	2.595	39.8	0.013

Continuous variables are reported as mean ± standard deviation (SD), and group differences were assessed with independent-samples t-tests, applying Welch’s correction when appropriate. Categorical variables (gender) are presented as counts and percentages, and group differences were assessed using Chi-squared tests (χ²). Autism Diagnostic Observation Schedule (ADOS); Degrees of freedom (df); Full-scale Intelligence Quotient (FIQ); Beck Depression Inventory (BDI)

### ALPS-index

We first examined the effects of diagnosis (ASD vs. NT) and age group (< 18 vs. ≥18 years) on the ALPS-index using a two-way ANOVA (Table 2). The analysis revealed a significant main effect of diagnosis, *F*(1, 246) = 10.44, *p* = 0.0014, η²ₚ = 0.041, indicating overall lower ALPS-indices in the ASD group compared to NT. A significant diagnosis × age group interaction was also observed, *F*(1, 246) = 4.71, *p* = 0.031, η²ₚ = 0.019, suggesting that the effect of diagnosis differed by age group. This indicates that the diagnostic effect (ASD vs. NT) varied across age groups (< 18 vs. ≥18 years), suggesting an age-dependent difference in ALPS-index.

**Table 2:** ALPS-index Two-way ANOVA and Post-hoc Comparisons

Effect ANOVA	F (1,246)		*P*		ηp²	
Diagnosis	10.440		0.0014		0.041	
Age Group	2.396		0.123		0.010	
Diagnosis × Age Group	4.714		0.031		0.019	

Age	Diagnosis	*N*	Mean ± SD	95% CI	ASD-NT Δ	t	*P* (post-hoc)	Cohen's D
<18	ASD	86	1.41 ± 0.19	[1.37, 1.45]	-0.030	-0.951	0.342	-0.16
	NT	65	1.44 ± 0.17	[1.40, 1.48]				
≥18	ASD	42	1.39 ± 0.20	[1.33, 1.45]	-0.140	-3.561	0.0004	-0.72
	NT	57	1.53 ± 0.22	[1.47, 1.59]				

The upper portion displays the two-way ANOVA results, including F-statistics, p-values, and Partial eta squared (ηp²) for the effects of diagnosis (ASD vs. NT), age group (<18 vs. ≥18 years), and their interaction (Diagnosis × Age Group). The lower panel provides ALPS-index group means ± standard deviation (SD) with 95% confidence intervals (CI), ASD-NT group differences (Δ), post-hoc p-values, and Cohen’s d effect sizes, where negative values indicate lower ALPS-index in ASD than NT

**Fig. 4:**
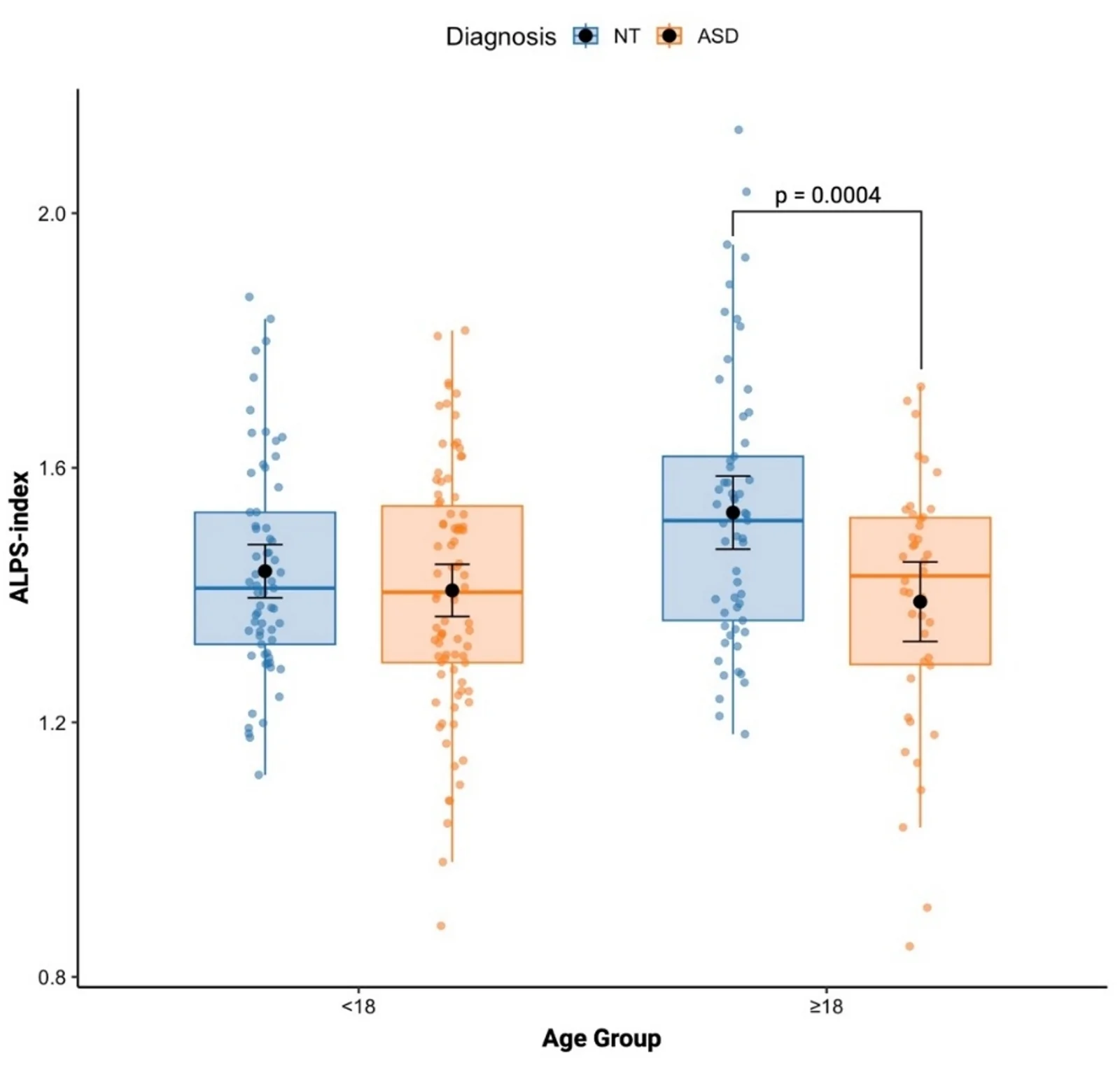
Group Differences in ALPS-index by Diagnosis and Age group. Boxplots show the distribution of ALPS-index values in neurotypical (NT, blue) and autistic (ASD, orange) participants stratified by age group (<18 years, ≥18 years). Individual data points are overlaid. Error bars indicate group means ± standard deviation. A significant group difference was observed in adults (≥18 years, *N* = 99), with lower ALPS-index in the ASD group compared to NT (*p* = 0.0004)

Post hoc pairwise comparisons showed no significant ALPS-index difference between ASD and NT participants in the young (< 18-year) group (*p* = 0.342, Cohen’s *d* = − 0.16, *N* = 151), whereas the adult (≥ 18-year) ASD group demonstrated significantly lower ALPS-indices compared with age-matched NT participants (*p* = 0.0004, Cohen’s *d* = − 0.72, *N* = 99) (Fig. 4). In contrast, no significant main effect of age group was detected in the ANOVA, *F*(1, 246) = 2.396, *p* = 0.123, η²ₚ = 0.010. Overall, these results indicate that the reduction in ALPS-index is primarily driven by autistic adults.

To address the potential limitations of age dichotomization, age was additionally modeled as a continuous variable. In this model, the diagnosis-by-age interaction was not significant in a linear specification (β = 0.002, *p* = 0.259), suggesting no linear age-dependent effect. However, when age was modeled nonlinearly using a generalized additive model, a significant diagnosis-by-age interaction was observed (*F = 2.545*, *p* = 0.0187,* η²ₚ = 0.06*), suggesting that the relationship between diagnosis and ALPS-index varies across age in a nonlinear manner. To assess the potential influence of confounding variables, we repeated the nonlinear modeling including FIQ, medication status, comorbidity, and scanner site as covariates in a generalized additive model. In this adjusted model, the diagnosis-by-age interaction was no longer significant (*F = 0.676*, *p* = 0.455), suggesting that the previously observed interaction is attenuated after accounting for these factors. Additionally, sensitivity analyses excluding medicated participants yielded comparable results (*F = 0.796*, *p* = 0.438). Similarly, excluding both medicated and comorbid participants yielded no significant diagnosis-by-age interaction *(F = 2.375*, *p* = 0.240). Exploratory stratified analyses within individual cohorts showed consistently non-significant diagnosis-by-age interactions across sites (all *p* > 0.223), indicating no evidence for a robust within-site effect.

### Correlation between ALPS-index and clinical measures

Given that FIQ differed significantly between ASD and NT groups (Table 1), we examined the relationship between the ALPS-index and FIQ. In the whole sample, no significant correlation was observed (*r* = − 0.060, *p* = 0.372). Stratified analyses revealed no significant correlations in either the ASD (*r* = − 0.101, *p* = 0.267) or NT group (*r* = − 0.037, *p* = 0.720). Similarly, no significant correlation was found when stratifying by age group (< 18 years: *r* = − 0.088, *p* = 0.294; ≥18 years: *r* = 0.014, *p* = 0.903). Fully stratified analyses by both diagnosis and age group also revealed no significant correlations (all *p* > 0.26), indicating that FIQ was not significantly correlated with ALPS-indices in any subgroup. Detailed results are provided in Supporting Information Table S2.

For the ASD group, we additionally assessed the association between ALPS-index and ADOS scores as a measure of the severity of core symptoms of autism. In the whole ASD sample, a weak negative, non-significant correlation was observed (*r* = − 0.161, *p* = 0.091). Subgroup analyses showed no significant associations in children/adolescents (< 18 years: *r* = 0.100, *p* = 0.390) or adults (≥ 18 years: *r* = 0.005, *p* = 0.975). We also examined the relationship between ALPS-index and depressive symptoms in adults using the BDI. BDI was only available in the Barrow cohort (*N* = 54), which only included adult participants (age range 18–64 years). In the whole adult sample, no significant association was observed (*r* = − 0.209, *p* = 0.129). However, when stratified by diagnosis, a significant negative correlation was identified in the adult ASD group (*r* = − 0.489, *p* = 0.013), whereas no significant correlation was found in NT adults (*r* = 0.005, *p* = 0.982). This suggests that the ALPS-index is largely unrelated to general cognitive ability (FIQ) or autism symptom severity (ADOS) in autistic individuals, whereas lower ALPS-indices in autistic adults show modest correlation with higher depressive symptom severity. Detailed correlation results are provided in Supporting Information Table S2.

## Discussion

In this cross-sectional study spanning five independent cohorts, we observed that the ALPS-index was reduced in autistic compared to NT individuals. Notably, this group difference was age-dependent within this cross-sectional sample. When stratifying the sample into younger (< 18 years) and adult (≥ 18 years) participants, significantly lower ALPS-index values were only detected in autistic adults compared to NT adults, whereas no significant differences emerged between autistic and NT participants in the younger group. These findings stand in contrast to prior studies focusing on young children, which consistently reported lower ALPS-index values in ASD. Li et al. (2022) examined 30 autistic and 25 NT children (mean age 3.9 years, range 2–6 years) and reported significantly reduced ALPS-index in the ASD group (Li et al. 2022). Similarly, Wang et al. (2025) studied a larger pediatric sample (178 participants, mean age 4.9 years, range 3–7 years) and likewise found decreased ALPS-index in autistic compared to NT children (Wang et al. 2025). Lastly, Tian et al. (2025) included 89 autistic and 37 NT children (mean age 3.6 years, range 2–6 years) and demonstrated decreased ALPS-index in ASD (Tian et al. 2025). In the present cross-sectional sample, the initially observed diagnosis-by-age interaction suggests that group differences may vary across age groups. This interpretation was supported by the nonlinear, but not the linear, model when age was treated as a continuous variable, indicating a potential nonlinearity in age-related effects. However, this interaction was attenuated after adjustment for relevant covariates, including comorbidity, medication status, and cohort, suggesting that the observed age-dependent pattern may partly reflect underlying group differences in clinical and demographic characteristics rather than a purely diagnosis-driven developmental effect. This is supported by the absence of significant diagnosis-by-age interactions within individual cohorts. This may partly reflect reduced statistical power in these stratified analyses, yet the consistent lack of such effects across sites, combined with the attenuation observed after covariate adjustment, suggests that the initially observed interaction is not robust and may be driven by between-site variability and confounding factors. Furthermore, the cross-sectional design and limited statistical power, particularly in subgroup analyses, may also have contributed to the observed pattern. The discrepancy between our findings and prior reports may be explained by differences in participant age. The young group in our study had a substantially higher mean age (11.4 years, range 5–17 years) and did not include children as young as those examined in the prior studies. This age difference may have limited our ability to detect early developmental irregularities in glymphatic function. Given that ASD is a neurodevelopmental condition, glymphatic alterations may be most pronounced in the early years and then attenuate during later childhood, reflecting either delayed maturation or compensatory mechanisms. Supporting this notion, several studies have reported abnormalities in the glymphatic system in young children with ASD (Shen et al. 2018; Garic et al. 2023). In line with a developmental trajectory, glymphatic function (as indexed by ALPS) has been shown to increase with age across early childhood and adolescence (Wong et al. 2025). Consistently, Li et al. (2022) reported a positive correlation between ALPS-index and age in both ASD and NT children (Li et al. 2022). More broadly, evidence from a large cross-sectional study in NT individuals suggests that ALPS-index values continue to rise until approximately 40 years of age, followed by a gradual decline thereafter (Taoka et al. 2022). ASD is associated with an atypical developmental trajectory of brain volume, characterized by early brain overgrowth followed by reduced gray matter volume later in development (Levman et al. 2018; Prigge et al. 2021), this pattern also appears to involve changes in the choroid plexus. Several studies have reported increased choroid plexus volume during childhood and adulthood in ASD, although the magnitude of enlargement varies across ages (Levman et al. 2018; Tezcan et al. 2025) similarly to our results. The choroid plexus is a specialized epithelium within the brain’s ventricles that produces CSF and regulates the composition and circulation of the fluid that drives the glymphatic clearance system. Because choroid plexus enlargement has been associated with reduced glymphatic function (Gong et al. 2025), this may indicate that the glymphatic system is consistently, but in varying degrees dependent on age, affected in ASD.

Taken together, these findings suggest that glymphatic function in ASD may change dynamically across development, with alterations most evident during specific age periods. However, longitudinal multimodal imaging studies are needed to clarify these developmental patterns and determine their relevance to the clinical and neurobiological features of autism.

### Glymphatic function in aging autistic individuals

In our study, glymphatic function, assessed using the ALPS-index, was reduced in autistic adults. Prior work has shown that the ALPS-index correlates positively with sleep quality, memory performance, and neural communication in older adults (Ma et al. 2025), underscoring the importance of preserved glymphatic function for healthy brain aging (Benveniste et al. 2019). If ASD is indeed associated with reduced glymphatic function, as suggested by our findings, this could provide a mechanistic link to the increased risk of AD reported in autistic individuals (Vivanti et al. 2021; Sian-Hulsmann et al. 2026). Supporting this interpretation, postmortem studies have identified greater accumulation of amyloid-β in autistic brains (Wegiel et al. 2012). Considering that amyloid-β accumulates with increasing age (Knudsen et al. 2025) and is cleared by the glymphatic system, enhanced accumulation could be caused by reduced glymphatic function (Iliff et al. 2012), as demonstrated rodent models.

Furthermore, middle-aged and elderly autistic individuals have been shown to exhibit more severe reductions in both memory performance and hippocampal volume (Pagni et al. 2022; Walsh et al. 2022), both of which are indicators of AD. Interestingly, recent studies have reported positive correlations between glymphatic function and hippocampal volume (Chang et al. 2023), suggesting that impaired glymphatic clearance may be associated with the AD-like decline in hippocampal volume and memory performance observed in autistic individuals later in life.

Another possible mechanism underlying reduced glymphatic function in ASD could be related to neuroinflammation and oxidative stress, which are frequently reported in autistic individuals (Thorsen et al. 2021; Than et al. 2023). Notably, both processes can damage astrocytic endfeet, causing a disruption of the AQP4 polarization, as well as vascular stiffening and impaired arterial pulsatility (Cai et al. 2024). These are all mechanisms that could contribute to reduced perivascular glymphatic function (Iliff et al. 2013; Cai et al. 2024).

Taken together, these converging lines of evidence suggest that altered glymphatic-related processes in ASD may be involved in both neurodevelopmental alterations and increase vulnerability to later-life neurodegenerative pathology. However, the underlying causes of reduced glymphatic function remains uncertain and glymphatic function was only assessed indirectly in this study using DTI-ALPS. Therefore, future work integrating longitudinal imaging, sleep assessment, measures of neuroinflammation, and biomarker studies will be essential to clarify the role of glymphatic dysfunction in linking autism with age-related neurological disorder.

### Correlation analyses

In our correlation analysis, we observed a significant negative association between depressive symptoms (BDI score) and ALPS-index. This is noteworthy given that individuals with ASD are at increased risk for depression (Hudson et al. 2019), and depression in ASD is frequently accompanied by sleep disturbances (Stewart et al. 2020; Puzino et al. 2021). Because sleep quality has been closely linked to glymphatic function (Saito et al. 2023; Kikuta et al. 2024; Ma et al. 2025), it is plausible that disrupted sleep is linked to altered glymphatic clearance in ASD. Moreover, decreased ALPS-index have been demonstrated in patients with major depressive disorder (Bao et al. 2025). Consequently, the high prevalence of depression and anxiety in autism may, at least in part, be associated with limited glymphatic function.

In contrast, we did not observe significant correlations between ALPS-index and either FIQ or autistic traits. This differs from the findings of Wang et al. (2025) and Tian et al. (2025) who both reported a negative correlation between ALPS-index and ASD severity measured by the Childhood Autism Rating Scale (CARS) (Schopler et al. 1980). Their result is consistent with prior studies linking autistic traits to sleep disturbances (Stewart et al. 2020). The discrepancy between our findings and prior studies may be explained by differences in study design, including our broader age range and the use of ADOS rather than CARS as the clinical measure.

### Limitations

There are several limitations of our study that are worth discussing. First, the ALPS-index is an indirect proxy of glymphatic function, derived from the ratio of water diffusivity along PVS to that measured perpendicular to the PVS at the level of the lateral ventricles (Taoka et al. 2017, 2024). As such, it primarily reflects left-right Brownian motion of water molecules in this region. However, waste clearance from the brain parenchyma also follows radial pathways toward the cortical surface and ventricular walls, which are not fully captured by this measure. Consequently, the ALPS-index provides only an indirect approximation of glymphatic function. In principle, a lower ALPS-index reflects slower water diffusivity along perivascular pathways, which may indicate reduced solute clearance capacity and, to some extent, impaired glymphatic function. However, it does not capture the full complexity of glymphatic function across the brain. Future work employing diffusion MRI with multiple b-values, and combining ALPS with complementary imaging approaches (e.g., advanced CSF flow imaging), would provide a more comprehensive understanding of glymphatic function in ASD.

Second, because our study is cross-sectional, we cannot directly assess longitudinal changes in glymphatic function across development or aging in ASD. Longitudinal studies spanning early childhood through adulthood will be necessary to establish developmental trajectories and clarify potential causal links with clinical outcomes.

Third, although the ALPS-index equation includes an inherent normalization factor, as it is the ratio of internal references (Taoka et al. 2017), and our cohort comparisons did not indicate scanner-related differences (Supporting Information Table S1 and Fig. S1), we cannot entirely exclude the possibility that data from different scanners influenced the results.

Fourth, while the overall sample size was relatively large, stratified subgroup analyses (e.g., adults only) reduced the effective sample size, which may have limited statistical power. Additionally, the limited number of female participants (*n* = 35) prevented us from assessing sex differences.

Fifth, although interstitial solute clearance occurs during wakefulness, the interstitial space expands by approximately 60% during sleep, markedly enhancing glymphatic flow (Xie et al. 2013). Thus, glymphatic function is most accurately assessed during sleep, whereas in our study participants were imaged at rest while awake. This limitation highlights the importance of considering sleep–wake state in future glymphatic research.

Sixth, we observed a significant difference in both comorbidity and medication use between groups, which was expected given the high prevalence of comorbidities in ASD (Simonoff et al. 2008). Nineteen participants in the ASD group had a comorbid condition (1 with anxiety, 18 with ADHD), whereas only one participant in the NT group had a comorbidity (thyrotoxicosis) (Fig. 3). Considering that approximately 40% of autistic individuals are diagnosed with ADHD (Rong et al. 2021), the comparatively lower prevalence of around 15% in our cohort suggests that our findings may not be fully generalizable to the broader autistic population. Accordingly, 25 participants in the ASD group reported medication use (2 amphetamine, 1 atomoxetine, 3 bupropion, 1 citalopram, 2 escitalopram, 1 fluoxetine, 2 lisdexamfetamine, 2 melatonin, 5 methylphenidate, 2 sertraline, 3 venlafaxine, and 1 ziprasidone), compared to only two participants in the NT group (1 levothyroxine and 1 antidepressant). How and whether these medications influence glymphatic function remains unknown and may therefore represent a potential source of bias.

Lastly, the age-dependent group differences observed in this study are based on cross-sectional data and therefore do not provide direct evidence of developmental change. Such patterns may also reflect cohort effects, differences in sampling across age groups, or other unmeasured confounders. Longitudinal studies will be required to disentangle these possibilities.

## Conclusions

In this multi-cohort cross-sectional study, we provide evidence suggestive of differences in ALPS-index values, an indirect marker of glymphatic function, in autistic individuals, with the effect most pronounced in adulthood. These findings indicate that glymphatic alterations in autism may vary across development, in a pattern that partly parallels the age-related trajectory of brain volume, although the cross-sectional design precludes conclusions about developmental trajectories. Reduced glymphatic function in autistic adults may help explain their increased vulnerability to memory decline, hippocampal atrophy, and AD pathology, while also linking to the high prevalence of depression and sleep disturbances in this population. Taken together, our results support the hypothesis that glymphatic dysfunction may represent a common mechanistic pathway linking the neurodevelopmental features of autism with an increased risk of psychiatric and neurodegenerative diseases later in life. However, the ALPS index is only an indirect measure of glymphatic function and the causes of reduced glymphatic function remain uncertain. Future longitudinal and multimodal studies will be essential to clarify the role of glymphatic function in autism across the lifespan and to determine its clinical relevance for both early development and age-related outcomes.

## Supplementary Information

Below is the link to the electronic supplementary material.

Supplementary Material 1

## Data Availability

Data is freely accessible at the Autism Brain Imaging Data Exchange: https://fcon_1000.projects.nitrc.org/indi/abide/.
