# Supplementary material for: Reduced glymphatic function in autism revealed by the diffusion tensor analysis along the perivascular space index
Source: J Neural Transm (Vienna). 2026 Aug 7;133(8):1857–69. doi: 10.1007/s00702-026-03249-2 (PMC13545022; doi:10.1007/s00702-026-03249-2)
Supplement: Supplementary file 1 [file 702_2026_3249_MOESM1_ESM.docx]

**Supplementary materials for Knudsen et al., Reduced Glymphatic Function in Autism Revealed by the Diffusion Tensor Analysis Along the Perivascular Space Index**

**CONTENTS**

**Table S1.** Cohort-wise descriptive statistics of ALPS-index values. **Figure S1.** Cohort-wise mean ALPS-index values. **Table S2.** Pearson correlations between the ALPS-index and cognitive/clinical measures.

The authors have provided this supplementary material to offer readers additional context and details related to their study.

**Supplementary Table S1. Cohort-wise descriptive statistics of ALPS-index values.**

| **Cohort** | **Group** | **N** | **ALPS-index** | | |
| --- | --- | --- | --- | --- | --- |
|  |  |  | **Mean** | **SD** | **CI** |
| Barrow | NT | 29 | 1.412 | 0.124 | 1.365-1.459 |
|  | ASD | 29 | 1.388 | 0.164 | 1.326-1.451 |
| Institute Pasteur | NT | 27 | 1.694 | 0.223 | 1.606-1.783 |
|  | ASD | 15 | 1.458 | 0.352 | 1.294-1.623 |
| NYU | NT | 22 | 1.353 | 0.111 | 1.304-1.402 |
|  | ASD | 33 | 1.326 | 0.165 | 1.267-1.384 |
| San Diego State | NT | 24 | 1.442 | 0.121 | 1.391-1.493 |
|  | ASD | 32 | 1.428 | 0.178 | 1.365-1.491 |
| Trinity | NT | 20 | 1.479 | 0.179 | 1.394-1.563 |
|  | ASD | 19 | 1.477 | 0.159 | 1.402-1.551 |

ASD = autism spectrum disorder, CI = confidence interval, NT = neurotypical, SD = Standard deviation.

**Supplementary Figure S1. Cohort-wise mean ALPS-index values.**

Mean ALPS-index values for each cohort, stratified by diagnosis. ASD = autism spectrum disorder, NT = neurotypical.

**Supplementary Table S2. Pearson correlations between the ALPS-index and cognitive/clinical measures.**

| **Measure** | **Group** | **N** | **r** | **p-value** | **Bonferroni corrected α** |
| --- | --- | --- | --- | --- | --- |
| **FIQ** | Whole sample | 222 | –0.060 | 0.372 | 0.0056 |
|  | ASD | 123 | –0.101 | 0.267 | 0.0056 |
|  | NT | 99 | –0.037 | 0.720 | 0.0056 |
|  | <18 years | 144 | –0.088 | 0.294 | 0.0056 |
|  | >18 years | 78 | 0.014 | 0.903 | 0.0056 |
|  | ASD <18 years | 82 | –0.125 | 0.264 | 0.0056 |
|  | ASD >18 years | 41 | –0.038 | 0.816 | 0.0056 |
|  | NT <18 years | 62 | –0.064 | 0.624 | 0.0056 |
|  | NT >18 years | 37 | 0.045 | 0.790 | 0.0056 |
| **ADOS** | ASD only | 117 | -0.161 | 0.091 | 0.0133 |
|  | ASD <18 years | 76 | 0.100 | 0.390 | 0.0133 |
|  | ASD >18 years | 41 | 0.005 | 0.975 | 0.0133 |
| **BDI** | >18 years | 54 | -0.209 | 0.129 | 0.0133 |
|  | NT >18 years | 29 | 0.005 | 0.982 | 0.0133 |
|  | ASD >18 years | 25 | -0.489 | 0.013 | 0.0133 |

Bonferroni correction applied separately for each clinical measure (FIQ: 9 tests, α = 0.05/9 = 0.0056; ADOS: 3 tests, α = 0.05/3 = 0.0133; BDI: 3 tests, α = 0.05/3 = 0.0133). Values in the last column indicate the corrected significance threshold, not adjusted p-values. Autism Diagnostic Observation Schedule (ADOS); Full-scale Intelligence Quotient (FIQ); Beck Depression Inventory (BDI).
